# Artificial neural network analysis of microbial diversity in the central and southern Adriatic Sea

**DOI:** 10.1038/s41598-021-90863-7

**Published:** 2021-05-27

**Authors:** Danijela Šantić, Kasia Piwosz, Frano Matić, Ana Vrdoljak Tomaš, Jasna Arapov, Jason Lawrence Dean, Mladen Šolić, Michal Koblížek, Grozdan Kušpilić, Stefanija Šestanović

**Affiliations:** 1 Institute of Oceanography and Fisheries, Šetalište Ivana Meštrovića 63, POB 500, 21000 Split, Croatia; 2 National Marine Fisheries Research Institute, Kołłątaja 1, 81-332 Gdynia, Poland; 3 Centre Algatech, Institute of Microbiology of the Czech Acad. Sci., 379 81 Třeboň, Czech Republic; 4 University of South Bohemia, Faculty of Science, Branišovská 1760, Ceske Budejovice, Czech Republic

**Keywords:** Biodiversity, Biooceanography, Community ecology, Ecological networks, Microbial ecology, Ecology, Molecular biology, Ecology, Environmental sciences, Ocean sciences, Mathematics and computing

## Abstract

Bacteria are an active and diverse component of pelagic communities. The identification of main factors governing microbial diversity and spatial distribution requires advanced mathematical analyses. Here, the bacterial community composition was analysed, along with a depth profile, in the open Adriatic Sea using amplicon sequencing of bacterial 16S rRNA and the Neural gas algorithm. The performed analysis classified the sample into four best matching units representing heterogenic patterns of the bacterial community composition. The observed parameters were more differentiated by depth than by area, with temperature and identified salinity as important environmental variables. The highest diversity was observed at the deep chlorophyll maximum, while bacterial abundance and production peaked in the upper layers. The most of the identified genera belonged to Proteobacteria, with uncultured AEGEAN-169 and SAR116 lineages being dominant Alphaproteobacteria, and OM60 (NOR5) and SAR86 being dominant Gammaproteobacteria. Marine *Synechococcus* and *Cyanobium*-related species were predominant in the shallow layer, while *Prochlorococcus* MIT 9313 formed a higher portion below 50 m depth. Bacteroidota were represented mostly by uncultured lineages (NS4, NS5 and NS9 marine lineages). In contrast, Actinobacteriota were dominated by a candidatus genus *Ca.* Actinomarina. A large contribution of Nitrospinae was evident at the deepest investigated layer. Our results document that neural network analysis of environmental data may provide a novel insight into factors affecting picoplankton in the open sea environment.

## Introduction

Microorganisms drive the biogeochemical fluxes in marine environments. Since various microbial processes are conducted by different species, knowledge of bacterial community composition (BCC) is essential to understand the functioning of a particular ecosystem [e.g.^1^]. Knowing that the BCC is closely associated with environmental conditions, many studies conducted in different marine ecosystems, including the Mediterranean Sea, have linked these characteristics using different scales^2–8^ and highlight the importance of interdependencies, particularly between free-living bacteria in the upper layers of the epipelagic zone^9^.

In our previous work we focused on the role of heterotrophic bacteria, picoautotrophs and aerobic anoxygenic phototrophic (AAP) bacteria in the production and transfer of biomass and energy through the microbial food web in the coastal and open Adriatic Sea^10–16^. The Adriatic Sea is a highly dynamic environment, where the picoplankton community is exposed to sudden physio-chemical changes^14^. Its open waters are generally phosphorus (P) and nitrogen (N) limited^17^, however they can occasionally be enriched with nutrients and organic substances from advected water masses, leading to changes in the food web structure^10,14^. Indeed, increased microbial diversity was found to be associated with deep winter convection in the South Adriatic Sea^18,19^. The herbivorous food web dominates during such periods of the nitrate-rich mixed water column, but it gradually changes to a multivorous food web, which in turn changes to the typical microbial food web during the stratified period^20^. Moreover, unusually high salinity values for this area have been recorded since 2016, which have been shown to adversely affect the picoplankton community^21^. In addition to the bacterial community research mentioned above, the recent use of Self-organizing map analysis has elucidated the response of the microbial food web to changes in seawater temperature^10^, as mentioned previously. Different artificial neural network methods have already been used successfully for several biological applications^10,22–24^.

We propose that the picoplankton community display a heterogenic response to changes associated with different environmental factors in the open sea areas of the central and southern Adriatic Sea, throughout the stratified water column. To test this hypothesis, we used a Neural gas algorithm to identify bacterial community response in terms of abundance and community composition. The survey was conducted in three open sea areas, the South Adriatic Pit and, for the first time, the Jabuka Pit and Palagruža Sill. The novelty of this study is community characterisation and its relation with environmental data using the Neural gas algorithm^25^. The Neural gas algorithm, without the use of prior knowledge about the topological structure of data, quantified the manifold by distributing neural units homogenously over the input space^26^.

## Material and methods

### Sampling sites

The Adriatic Sea is an elongated semi-enclosed basin of the Eastern Mediterranean Sea, connected with the Ionian Sea through the Strait of Otranto [e.g.^18,27^]. According to its morphology and bathymetry, it is divided into three sub-basins (northern, central and southern). Jabuka Pit (JP) is located in the central Adriatic, with depths up to approximately 270 m. Palagruža Sill (PS) (170 m deep) separates the Jabuka Pit from the much deeper South Adriatic Pit (SAP) (1250 m deep).

**Figure 1:**
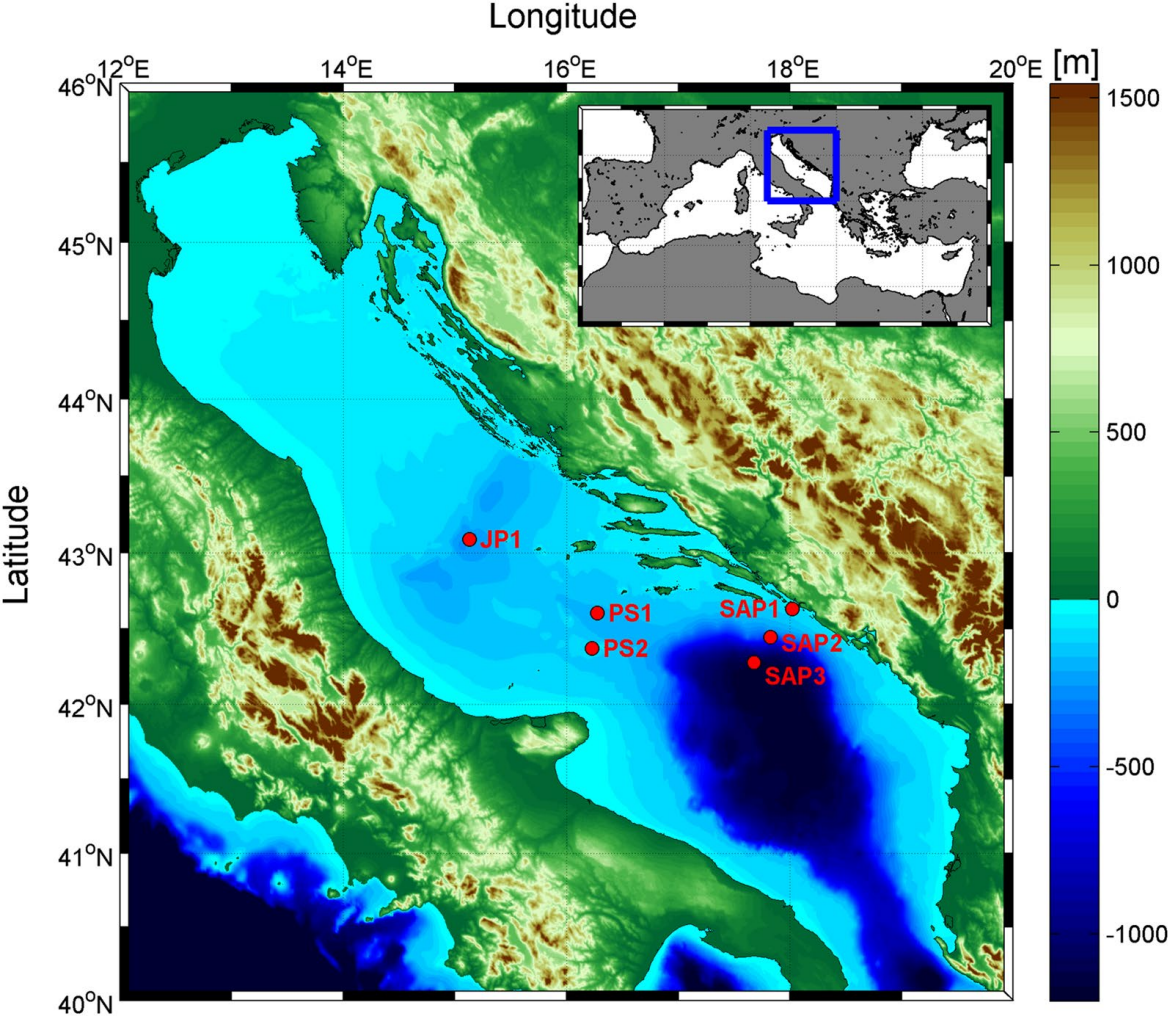
(**A**) Map of the Mediterranean Sea. (**B**) Study area and sampling stations in the Jabuka Pit (JP1), the Palagruža Sill (PS1, PS2) and the South Adriatic Pit (SAP1, SAP2, SAP3). The software MATLAB. version 7.10.0 (R2018). Natick, Massachusetts: The MathWorks Inc. (2018) (https://www.mathworks.com/) was used to generate the figure.

In total, 43 samples (from the surface to the bottom) were collected from 6 sites located in the area of Jabuka Pit (JP1), Palagruža Sill (PS1, PS2) and the South Adriatic Pit (SAP1, SAP2, SAP3) during September/October 2016 (29.09.-03.10) (Fig. 1).

### Environmental parameters

Temperature and salinity were measured using a SeaBird 25 CTD profiler with accuracy > ± 0.01 °C and ± 0.02, respectively. Concentrations of inorganic nutrients were determined using the autoanalyser-modified method^28^. Detection limits were 0.001 μmol L^−1^ for NO_2_^−^, 0.01 μmol L^−1^ for NO_3_^−^, 0.0002 mg L^−1^ for NH_4_^+^ and 0.02 μmol L^−1^ for soluble reactive phosphorus (SRP).

### Criteria for nutrient limitation

Nutrient limitation at the studied sites was assessed based on stoichiometry defined in^29^. Microorganisms in samples with concentrations of SRP < 0.1 μmol L^−1^, and with N/P ratio > 22 and Si/P ratio > 22 were considered to be limited by phosphorus. The criteria for nitrogen limitation was total inorganic nitrogen (TIN) < 1 μmol L^−1^^29^.

### Microbial analysis

#### Sample collection, DNA extraction and sequencing

Samples for DNA sequencing were taken at various depths based on physico-chemical parameters: euphotic zone, oxygen concentration maximum, chlorophyll *a* maximum and deep layers. These conditions were different for each station: SAP1: 40 m; SAP2: 5 m, 20 m and 33 m; SAP3: 5 m, 20 m, 78 m and 100 m; JP1: 0 m, 5 m, 10 m, 20 m, 30 m, 50 m, 66 m and 75 m; PS1: 5 m, 25 m and 65 m; and PS2: 5 m, 16 m and 50 m. 0.5–1 L of seawater was filtered through 0.22 µm polycarbonate filters (Merck Millipore, USA). The filters were flash-frozen in liquid nitrogen and stored at − 80 °C for < 1 month. DNA was extracted using the Power Water DNA isolation kit (MO BIO Laboratories, Inc., Carlsbad, CA, USA).

The V3-V4 region of the 16S rRNA gene was amplified using 341F-785R primers^30^ and Phusion HotStart II High Fidelity PCR MasterMix (Thermo Scientific). PCR conditions were the following: initial denaturation for 3 min at 98 °C, 25 cycles of 98 °C for 10 s, 60 °C for 20 s, 72 °C for 20 s, final elongation at 72 °C for 5 min. The amplicons were purified from the gel using Wizard SV Gel and PCR clean system (Promega) and quantified with Qubit dsDNA HS assay. Library preparation (2 × 250 bp) and sequencing on a MiSeq Illumina were performed by the Genomic Service of the Universitat Pompeu Fabra, Barcelona, Spain. The number of raw reads per sample ranged from 74,051 to 193,832 (mean ± st. dev. 100,931 ± 24,420).

#### Sequence analysis

The quality of reads was evaluated using FastQC v0.11.7. The sequences of the primers were trimmed using cutadapt v1.16^31^ and subsequent analyses were carried out in the R/Bioconductor environment using the dada2 v1.6 package^32^. Low quality sequences were filtered out with the filterAndTrim function (maxN = 0 (no N allowed), maxEE = c (2, 2), truncQ = 2, rm.phix = TRUE). After quality filtering and denoising, the number of reads per sample ranged from 70,750 to 187,751 (mean ± st. dev. 96,216 ± 23,563). After error learning and sequence variants interference, the sequences were merged, and the amplicon sequence variant (ASV) table was produced. Chimeric sequences were removed using the default removeBimeraDenovo function with the “consensus” method. The taxonomic assignment was performed with the naive Bayesian classifier method using the SILVA 132 database^33,34^. The final ASV table contained from 68,483 to 185,399 reads per sample (mean ± st. dev. 93,317 ± 23,087). The total number of observed ASVs was 4102, and it ranged from 391 to 1426 in specific samples. Removal of non-bacterial ASVs, ASVs classified as chloroplasts or mitochondrion, and those seen less than three times in at least 20% of the samples reduced the number of ASVs from 4102 to 714 (252–454 per sample), and the number of reads to 1,712,027. The phyloseq v1.30 package^35^ was used for this operation. Figures displaying characteristic genus and phylum contribution of the bacterial community composition were generated in R statistical software^36^ (https://cran.r-project.org/, v. 3.6.3), using ggplot2 v. 3.3.3 package^37^, and they were assembled in Inkscape (https://inkscape.org/, v. 0.92).

#### Flow cytometry

Picoplankton abundance was determined using flow cytometry^38^. For the count of autotrophic cells, 2 mL of sample were fixed with glutaraldehyde (0.5% final concentration) and stored at − 80 °C until analysis (5–10 days). Autotrophic cells were classified into three groups: *Synechococcus*, *Prochlorococcus* and picoeukaryotes, distinguished according to light scattering, red emission of cellular chlorophyll content and orange emission of phycoerythrin-rich cells. Samples for bacteria and heterotrophic nanoflagellate (HNF) abundance were fixed with formaldehyde (2% final concentration) and stored at 4 °C until analysis (5–10 days). They were stained with Sybr Green-I, and three groups, namely, high nucleic acid content (HNA) bacteria, low nucleic acid content (LNA) bacteria and HNF were determined using flow cytometry^38^. The samples were processed on a Beckman Coulter EPICS XL-MCL flow cytometer with a high flow rate from 1 to 1.2 μL s^−1^, and the analysed volume was calculated according to the acquisition time.

#### Epifluorescence microscopy

Aerobic Anoxygenic Phototrophs (AAP) were fixed with formaldehyde (0.5% final concentration) and stored at − 80 °C. AAP cells were collected on 0.2 µm polycarbonate filters by filtration and stained with 4′,6-diamidino-2-phenylindole (DAPI, 1 µg mL^−1^ final concentration) using a 3:1 mixture of Citifluor AF1 and Vectashield after drying^39^. AAP were counted using an Olympus BX51 microscope equipped with an Olympus UPlanSApo 100×/1.40 OIL, IR objective and software for image analysis (CellSens). The microscope was equipped with a Hg Lamp U-LH100H6 for excitation. Fluorescent images were taken using an XM10-IR camera. Three epifluorescent filter sets were used: DAPI, IR and red. The chlorophyll *a* signal was subtracted from the IR image to obtain a net count of AAP cells.

#### Bacterial production

Bacterial cell production was estimated by measuring the incorporation of ^3^H-thymidine into bacterial DNA^40^, which was added to 10 mL samples at a final concentration of 10 nmol (specific activity: 86 Ci mmol^–1^). Triplicate samples and a formaldehyde-killed absorption control (0.5% final concentration) were incubated for 1 h. The incubations were stopped with formaldehyde (0.5% final concentration). The thymidine samples were extracted with ice-cold trichloroacetic acid (TCA). The TCA-insoluble fraction was collected on 0.2 μm pore size polycarbonate filters. Samples were treated with Cocktail Filter Count (Perkin Elmer) before analysis on a liquid scintillation analyser TriCarb 4910 TR (Perkin Elmer).

### Statistical analysis

#### Calculation of diversity and evenness

The PRIMER 6 software package^41^ was used to calculate diversity indices: Shannon (H′) and Pielou's (J′), based on the untransformed and not rarefied ASV table.

#### Neural gas

Neural gas is an artificial neural network trained by unsupervised learning^26,42^. The method reduces the dimensionality of the data space to a certain number of neurons (Best-Matching Units, BMUs) distributed in the data space like a gas. During the learning process in a predefined number of adaptation steps together with the size of the adaptation step and neighbourhood impact range, the neural gas algorithm quantifies the manifold by distributing the BMUs over the relevant part of the input data space and tries to minimize the representation error. As a result, the connections between BMUs are weak without a predefined topological structure in the manifold. The lack of a fixed topological structure is the main difference from the commonly used Self-organizing maps that maximize the similarity between the BMUs and the measured data^10,20,22^. Weak connections between BMUs make neural gas a weak smoothing algorithm and more suitable for detecting anomalies and outlier data.

During the adaptation process, the neural gas was trained with 500 training epochs, with default values for initial step size (0.5) and an initial decay constant (2.5), following Martinetz et al.^25^. The number of BMUs was chosen to be 5 using a coefficient of variation of the SSIntra quantization error^43–45^. The data are scattered in PC1-PC2 space, making Principal Component Analysis (PCA) unsuitable for our analysis (Supplementary Fig. S1). The SOM-BMU distribution are always grouped in the origin and form more/less regular rectangular distribution, missing to model the data distant from centre. The Neural gas spreads enough to include all the data. As a result, the one extreme data is modelled with one BMU.

Two different NG analyses were conducted, picoplankton community (PIC) analysis and BCC. In the PIC-BMU analysis, the elements of the data vectors were separately normalized biotic parameters (total prokaryotes, *Synechococcus*, *Prochlorococcus*, picoeukaryotes, heterotrophic nanoflagellates, aerobic anoxygenic phototrophs, high and low nucleic acid bacteria, bacterial production), while in the BCC-BMU analysis they were separately normalized relative abundances of genera (bacterial community composition) obtained from sequencing. Both models were run using 22 different data vectors as sampling stations. The results of the analysis were PIC-BMU/BCC-BMU neural gas clusters. SOM Toolbox version 2.0 for MATLAB used in this study was developed by E. Alhoniemi, J. Himberg, J. Parhankangas, and J. Vesanto at Helsinki University of Technology, Finland, and is available at http://www.cis.hut.fi/projects/somtoolbox.

## Results

### Physico-chemical conditions

Sampling was performed at 6 stations representing the physical and chemical characteristics of the investigated area (Supplementary Table S1). Thermohaline properties were the result of horizontal advection of above-average salinities driven by a North Ionian cyclonic gyre controlled by the Adriatic Ionian Bimodal Oscillating System^46^. September and the whole summer of 2016 was characterized by extremely high temperatures, and precipitation in the climatologic expected range. A cyclone with a cold front followed by a strong Bora wind passed over the Adriatic a week before the cruise, in the period between the 16th and 20th of September 2016. Heat and mass exchange in the air-sea boundary layer were responsible for the characteristic vertical thermohaline profiles measured in late summer. Over the investigated area, the mixed layer depth located between 20 and 25 m was horizontally homogenous. The coldest water mass (temperature 12.94 °C, salinity 38.68) was located at the bottom of Jabuka Pit.

### Abundance of bacteria, autotrophic picoplankton and AAP

Bacterial abundances ranged between 0.05 and 0.46 × 10^6^ cell mL^−1^ in all three areas, with a slightly higher average value in Jabuka Pit (0.31 × 10^6^ cell mL^−1^). The bacterial abundances were the highest in the upper layers down to the 50 m deep layer and showed a decreasing trend towards the bottom (Supplementary Table S2). The portion of HNA bacteria ranged from 37.8 to 73.12% (on average 51.27%), with the prevalence of HNA over the LNA group below the epipelagic layer.

Marine *Synechococcus* dominated the autotrophic picoplankton community with abundances ranging from 0.08 to 23.86 × 10^3^ cell mL^−1^. The presence of *Prochlorococcus* cells was also detected in all samples in a range from a few cells to 1.33 × 10^3^ cell mL^−1^. Picoeukaryotes also showed a similar range from a few cells to 0.83 × 10^3^ cell mL^−1^. The highest abundances of picophytoplankton were measured in the upper 50 m, with the exception of the Palagruža Sill (PS) area, where an increase in abundance was observed at 100 m depth. Bacterial production ranged from 0.2 × 10^4^ to 0.36 × 10^4^ cell mL^−1^ h^−1^, with increased values in the shallow layers and a mostly uniform vertical distribution in the water column (Supplementary Table S2).

AAP bacteria abundance ranged from 0.9 × 10^3^ to 22.3 × 10^3^ cell mL^−1^, thus constituting 0.42% to 6.83% of the bacteria. Their highest average contribution was observed in the South Adriatic Pit (4.11%), while on the vertical scale, their highest contribution was observed in the upper 20 m of the seawater (see Supplementary Table S2).

### Relationship between the picoplankton community and environmental parameters

**Figure 2:**
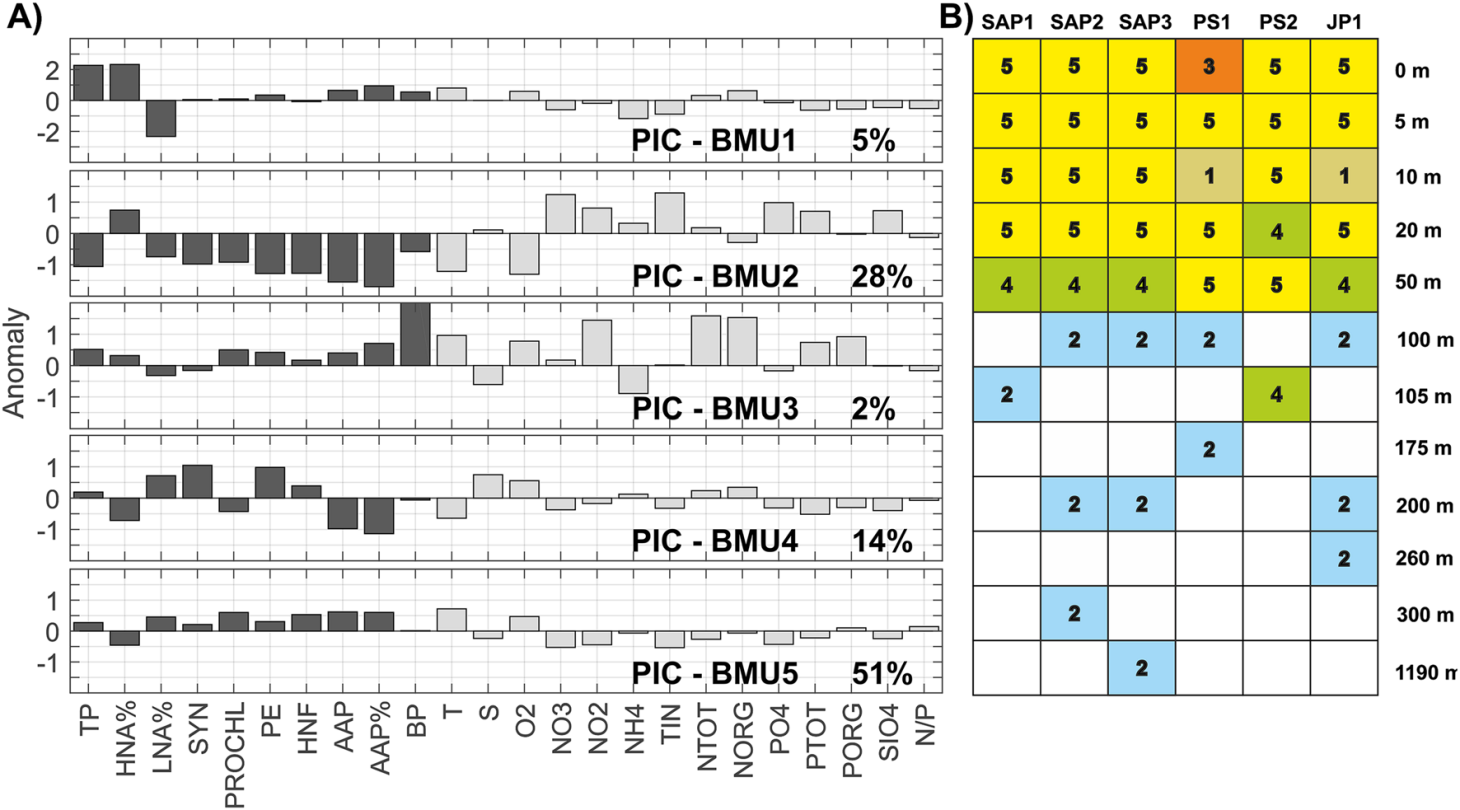
(**A**) Bar plot representation of biotic (black) and abiotic (grey) parameters for neural gas best-matching units (picoplankton-PIC-BMUs) with relative frequency appearance for each neuron. TP-total prokaryotes, SYN-*Synechococcus*, PROCHL-*Prochlorococcus*, PE-picoeukaryotes, HNF-heterotrophic nanoflagellates, AAP-aerobic anoxygenic phototrophs, AAP%-portion of AAP, HNA% percentage of high nucleic acid content bacteria, LNA%-percentage of low nucleic acid content bacteria-LNA%, BP-bacterial production. (**B**) Water column distribution of Neural gas best-matching units (BMU, labels with numbers, and stained with a different colour for clearance, coloured non-labelled squares shows clarity) for measuring stations (SAP1-3, PS1-2 and JP1). The software MATLAB. version 7.10.0 (R2018). Natick, Massachusetts: The MathWorks Inc. (2018) (https://www.mathworks.com/) was used to generate the figure.

Based on biological characteristics (total prokaryotes, *Synechococcus*, *Prochlorococcus*, picoeukaryotes, heterotrophic nanoflagellates, aerobic anoxygenic phototrophs, high and low nucleic acid bacteria, bacterial production), we distinguished five picoplanktonic clusters (PIC-BMUs) and then searched for explanations of the observed patterns (Fig. 2A,B). The mean values of biological and physico-chemical parameters for each cluster are shown in Table 1.

**Table 1:** Characteristics of biological (abundances of total prokaryotes-TP, *Synechococcus*-SYN, *Prochlorococcus*-PROCHL, picoeukaryotes-PE, heterotrophic nanoflagellates-HNF, aerobic anoxygenic phototrophs(AAP); contributions (%) of AAP, High nucleic acid content bacteria-HNA and Low nucleic acid content bacteria-LNA%; and bacterial production-BP) and environmental factors in the sampling terms assigned to the neural gas clusters.

	Neural gas clusters				
	PIC-BMU1	PIC-BMU2	PIC-BMU3	PIC-BMU4	PIC-BMU5
TP (10^6^ cell mL^−1^)	0.45 ± 0.02	0.14 ± 0.06	0.28	0.25 ± 0.09	0.26 ± 0.06
HNA (%)	65.39 ± 11.17	54.05 ± 5.54	51	43.62 ± 6.26	45.46 ± 3.38
LNA (%)	34.61 ± 11.17	45.95 ± 5.54	49	56.38 ± 6.23	54.54 ± 3.38
SYN (10^3^ cell mL^−1^)	6.77 ± 2.01	0.68 ± 0.62	5.51	12.58 ± 4.07	7.68 ± 5.80
PROCHL (10^3^ cell mL^−1^)	1.24 ± 0.31	0.36 ± 0.21	1.59	0.78 ± 0.64	1.68 ± 0.81
PE (10^3^ cell mL^−1^)	0.85 ± 0.08	0.27 ± 0.25	0.87	1.07 ± 0.20	0.83 ± 0.20
HNF (10^3^ cell mL^−1^)	0.86 ± 0.33	0.33 ± 0.17	0.97	1.06 ± 0.49	1.13 ± 0.24
AAP (10^4^ cell mL^−1^)	1.53 ± 0.31	0.18 ± 0.17	1.38	0.53 ± 0.42	1.51 ± 0.36
AAP (%)	5.79 ± 0.07	0.83 ± 0.66	5.34	1.89 ± 0.91	5.15 ± 0.73
BP (10^4^ cell h^−1^ mL^−1^)	0.10 ± 0.05	0.04 ± 0.01	0.36	0.06 ± 0.03	0.07 ± 0.02
Temperature (°C)	22.03 ± 0.62	14.43 ± 1.02	22.62	16.57 ± 1.37	21.70 ± 2.16
Salinity	38.81 ± 0.15	38.82 ± 0.10	38.75	38.87 ± 0.05	38.78 ± 0.08
O_2_ (%)	113.44 ± 5.42	96.21 ± 2.84	115.16	113.12 ± 11.56	112.35 ± 4.05
NO_3_^−^ (μmol L^−1^)	0.21 ± 0	1.77 ± 0.93	0.86	0.40 ± 0.47	00.20.27 ±
NO_2_^−^ (μmol L^−1^)	0.06 ± 0.01	0.12 ± 0.09	0.16	0.06 ± 0.02	0.05 ± 0.03
NH_4_^+^ (μmol L^−1^)	0.12 ± 0.10	0.48 ± 0.23	0.19	0.44 ± 0.24	0.39 ± 0.25
TIN (μmol L^−1^)	0.38 ± 0.09	2.37 ± 0.84	1.21	0.89 ± 0.39	0.70 ± 0.36
N tot (μmol L^−1^)	8.48 ± 3.22	8.13 ± 1.40	11.59	8.26 ± 3.16	7.02 ± 2.62
N org (μmol L^−1^)	8.09 ± 0.04	5.75 ± 0.06	10.38	7.37 ± 0.03	6.32 ± 0.02
SRP (μmol L^−1^)	0.05 ± 0.03	0.11 ± 0.08	0.05	0.04 ± 0.02	0.04 ± 0.05
P tot (μmol L^−1^)	0.13 ± 0.07	0.22 ± 0.07	0.22	0.14 ± 0.04	0.16 ± 0.06
P org (μmol L^−1^)	0.08 ± 0.06	0.11 ± 1.29	0.17	0.10 ± 0.22	0.12 ± 0.32
SiO_4_^−^ (μmol L^−1^)	0.91 ± 0.06	1.85 ± 1.29	1.27 ± 0.01	0.96 ± 0.22	1.08 ± 0.32

Mean values ± standard deviation from all the samples cluster in a PIC-BMU are displayed.

PIC-BMU1 described a very rare pattern, found in only two samples from 10 m depth in Palaguža Sill and Jabuka Pit. They were characterised by the highest abundances of total prokaryotes with a dominance of HNA and elevated AAP abundance. These samples were unique in terms of hydrological parameters, as they represented an N-limited environment (TIN < 1 µmol L^−1^, TIN/SRP < 10, Si/TIN > 1), where the water temperature was high (22.02 °C).

PIC-BMU2 described 28% of the samples. This cluster included the picoplankton community from the water column at and below 100 m and is characterised by the dominance of HNA in total prokaryote abundance and a decrease of all other picoplankton parameters. This layer describes the lowest seawater temperature (14.42 °C) and the highest concentrations of nitrates, nitrites, ammonium ions, silicates and SRP.

PIC-BMU3 included only one surface sample from Palagruža Sill with the highest values of bacterial production and AAP portion. The measured temperature was 22.62 °C and the highest values of N-organic compounds were detected in this sample. This sample was P-limited.

PIC-BMU4 described the pattern mostly from 50 m, with the highest contribution of LNA to total prokaryote abundance, the highest abundances of *Synechococcus* and picoeukaryotes, and the lowest AAP abundance. These samples were collected below the well-developed thermocline in the area of deep chlorophyll maximum (DCM) and were characterised by a P-limited environment with elevated salinity values compared to the other BMU clusters.

PIC-BMU5 described the most frequent pattern in our samples, the frequency can be attributed to the sampling effort rather than to certain general features of the sampling area. It grouped the samples from the first 50 m with high values for all measured biological parameters, except for HNA contribution to total prokaryote abundance. This layer is characterised by a temperature of approximately 21.70 °C and P limitation.

The results of the above analyses (Fig. 2A,B) document that all observed parameters were differentiated by depth rather than location. For at least 81% of the data, the negative impact of salinity is visible through the opposite direction of its anomaly values compared to most other picoplankton variables (heterotrophic bacteria, HNA%, LNA%, *Synechococcus*, *Prochlorococcus*, picoeukaryotes, AAP, bacterial production). Finally, our results suggest that an increase in temperature had a positive impact primarily in terms of high picoplankton abundances and bacterial productivity, given that the anomaly values of total prokaryotes, bacterial production and temperature display the same direction.

### Bacterial community composition

**Figure 3:**
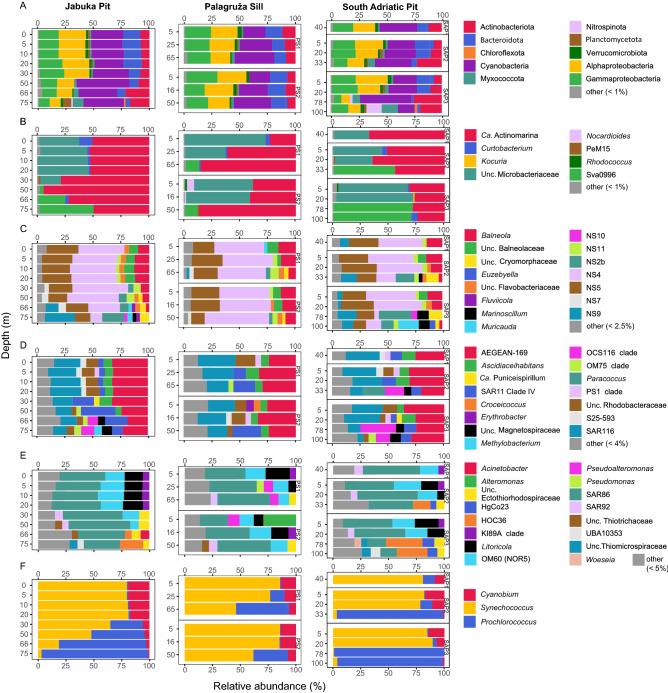
Bacterial community composition (BCC) in the Jabuka Pit (JP), Palagruža Sill (PS) and South Adriatic Pit (SAP). For the most abundant phyla or classes BCC as the genus level is shown. (**A**) Relative contribution of the bacterial phyla and proteobacterial classes (**B**) Genus contribution in the Actinobacteriota (**C**) Genus contribution in the Bacteroidota (**D**) Genus contribution in the Alphaproteobacteria (**E**) Genus contribution in the Gammaproteobacteria (**F**) Genus contribution in the Cyanobacteria. Category ‘other’ groups taxa with the relative abundances below the threshold given in the parentheses. The figure was generated in R statistical software^35^ (https://cran.r-project.org/ , v. 3.6.3), using ggplot2 v. 3.3.3 package^36^, and they were assembled in Inkscape (https://inkscape.org/ , v. 0.92).

Proteobacteria (mainly Alpha- and Gammaproteobacteria) and Cyanobacteria were the most abundant phyla in all samples, followed by Bacteroidota and Actinobacteriota. The changes between sites and depths at phylum level were minimal, with higher relative abundances of Planctomycota and Myxococcota observed at Jabuka Pit at 75 m, and of Nitrospinota and Myxococcota in the South Adriatic Pit at 100 m (Fig. 3A). The changes in bacterial communities were more conspicuous with depth rather than between the different basins, as will be shown below at the genus level for the most abundant phyla.

‘*Ca.*’ Actinomarina and uncultured Microbacteriaceae (Actinobacteriota) co-dominated in the upper layers down to about 20 m (Fig. 3B). The relative abundance of Microbacteriaceae substantially decreased in deeper layers, where it was replaced either by *‘Ca.’* Actinomarina or by uncultured Microtrichaceae from the Sva0996 lineage. The latter dominated in the deepest layers at the South Adriatic Pit (Fig. 3B).

Uncultured NS4 and NS5 lineages of Bacteroidetes co-dominated up to approximately 50 m depth, with minor contributions of genus *Balneola* and uncultured Balneolaceae, Cryomorphaceae and, especially in the Jabuka Pit, Flavobacteriaceae (Fig. 3C). Uncultured lineages NS2b and NS9 became relatively more abundant in deeper layers, together with genera *Marinoscillum* and *Muricauda.*

Proteobacteria was the most diverse phylum in terms of the number of detected genera. Thus, Alphaproteobacteria and Gammproteobacteria are shown separately (Fig. 3D,E, respectively). The members of Alphaproteobacteria with the highest relative abundance in the euphotic zone (~ 20 m) in all basins were affiliated with uncultured AEGEAN-169 and SAR116 lineages, with minor contributions of *Ascidiaceihabitans*, SAR11 clade IV, uncultured Rhodobacteraceae and S25–593 lineage. Uncultured Magnetospiraceae, *Methylobacterium* and OCS116 clade were more abundant in deeper layers (Fig. 3D). Moreover, 29 of genera with a relative abundance < 4% contributed 10–25% of all reads in all the samples.

The most abundant Gammaproteobacterial lineages in the upper layers down to about 50 m were OM60 (NOR5) and SAR86, with a minor contribution of KI89A clade and genus *Litoricola* (Fig. 3E). The exception was 5 m depth at station PS2, Palagruža Sill, where a higher relative abundance of *Alteromonas* was observed. Higher relative abundances of uncultured HOC36 and HgCo23lineages, and Ectothiorhodospiraceae and Thiomicrospiraceae were observed below 75 m. Moreover, a substantial proportion of Gammaproteobacterial reads consisted of numerous other genera with individual contributions < 5% (Fig. 3E). Cyanobacteria were represented by only three genera, with distinct distribution at different depths. Marine *Synechococcus* and *Cyanobium-like* sequences dominated in the euphotic zone, while *Prochlorococcus* in deeper waters (Fig. 3F).

**Figure 4:**
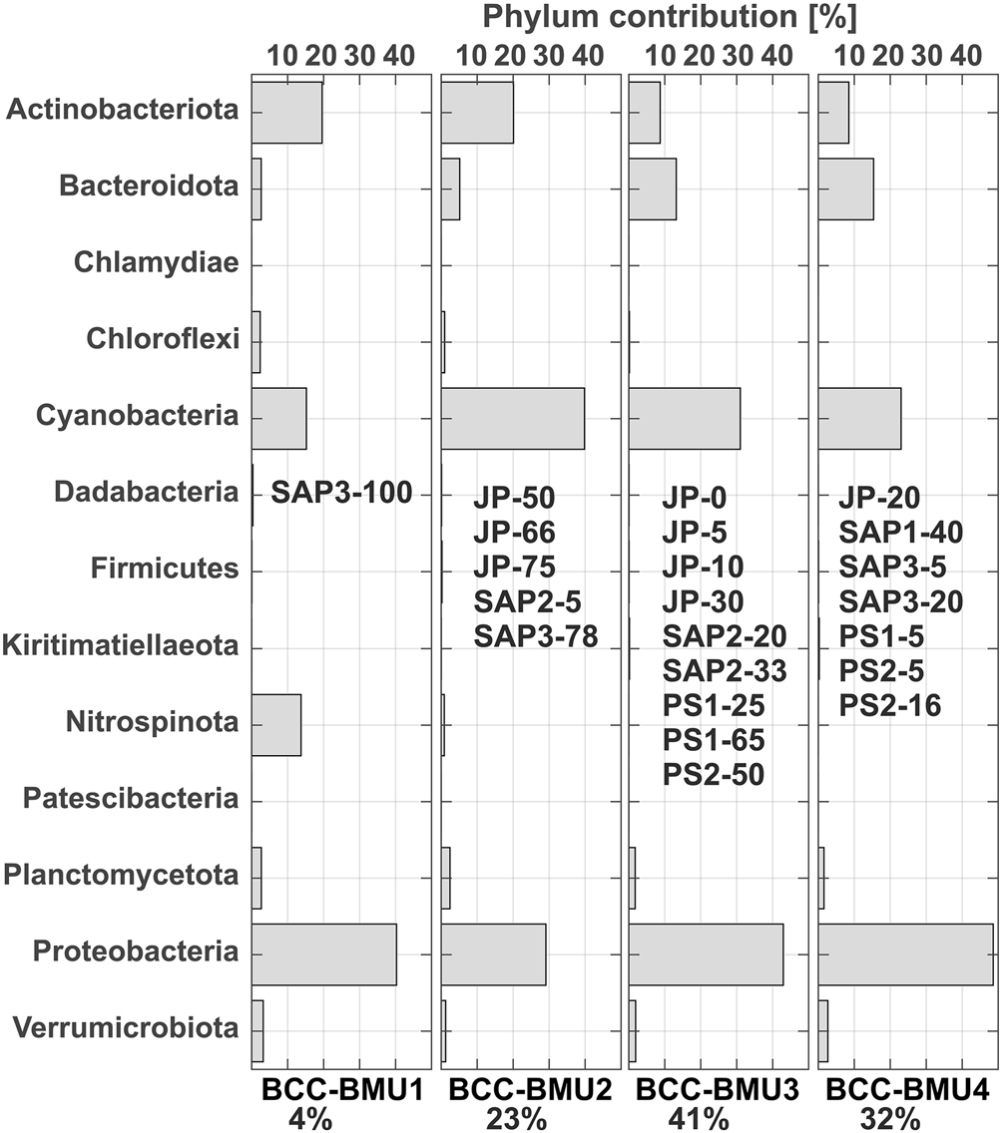
Characteristic phylum contribution (bacterial community composition-BCC-BMUs) modelled using Neural gas. Samples associated with a particular BMU are shown in the panels. The software MATLAB. version 7.10.0 (R2018). Natick, Massachusetts: The MathWorks Inc. (2018) (https://www.mathworks.com/) was used to generate the figure.

The neural gas analysis grouped the samples into four best matching units (BCC-BMU1, BCC-BMU2, BCC-BMU3, BCC-BMU4) representing heterogenic patterns of bacterial community composition that differed already at the phylum level (Fig. 4). Moreover, genera that showed higher importance in the samples were not always important for delineation of the BCC-BMU. The samples are grouped by depth rather than by basin.

**Figure 5:**
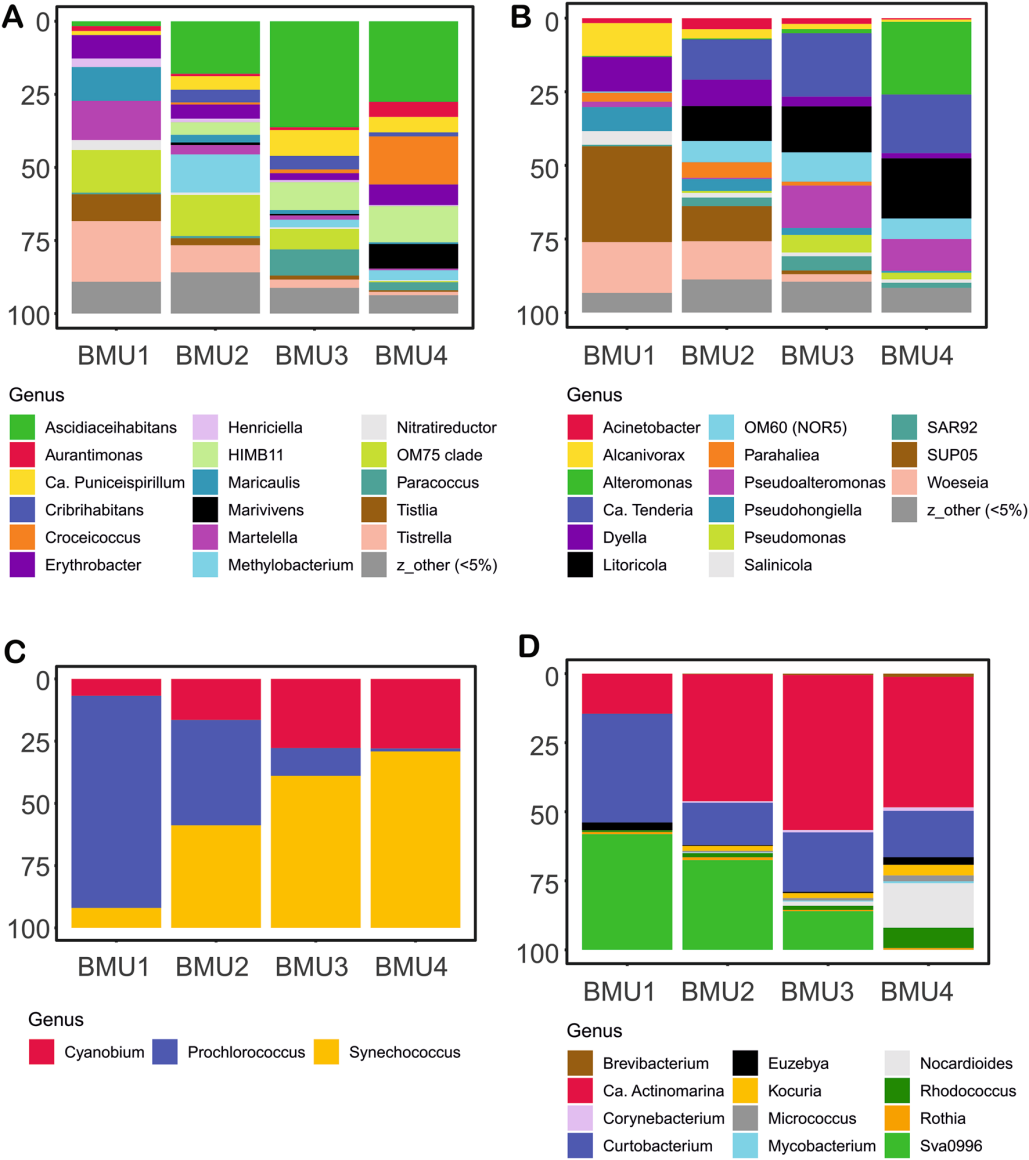
Characteristic genus contribution (**A**) Alphaproteobacteria (**B**) Gammaproteobacteria (**C**) Cyanobacteria (**D**) Actinobacteriota. The figure was generated in R statistical software^35^ (https://cran.r-project.org/ , v. 3.6.3), using ggplot2 v. 3.3.3 package^36^, and they were assembled in Inkscape (https://inkscape.org/ , v. 0.92).

**Figure 6:**
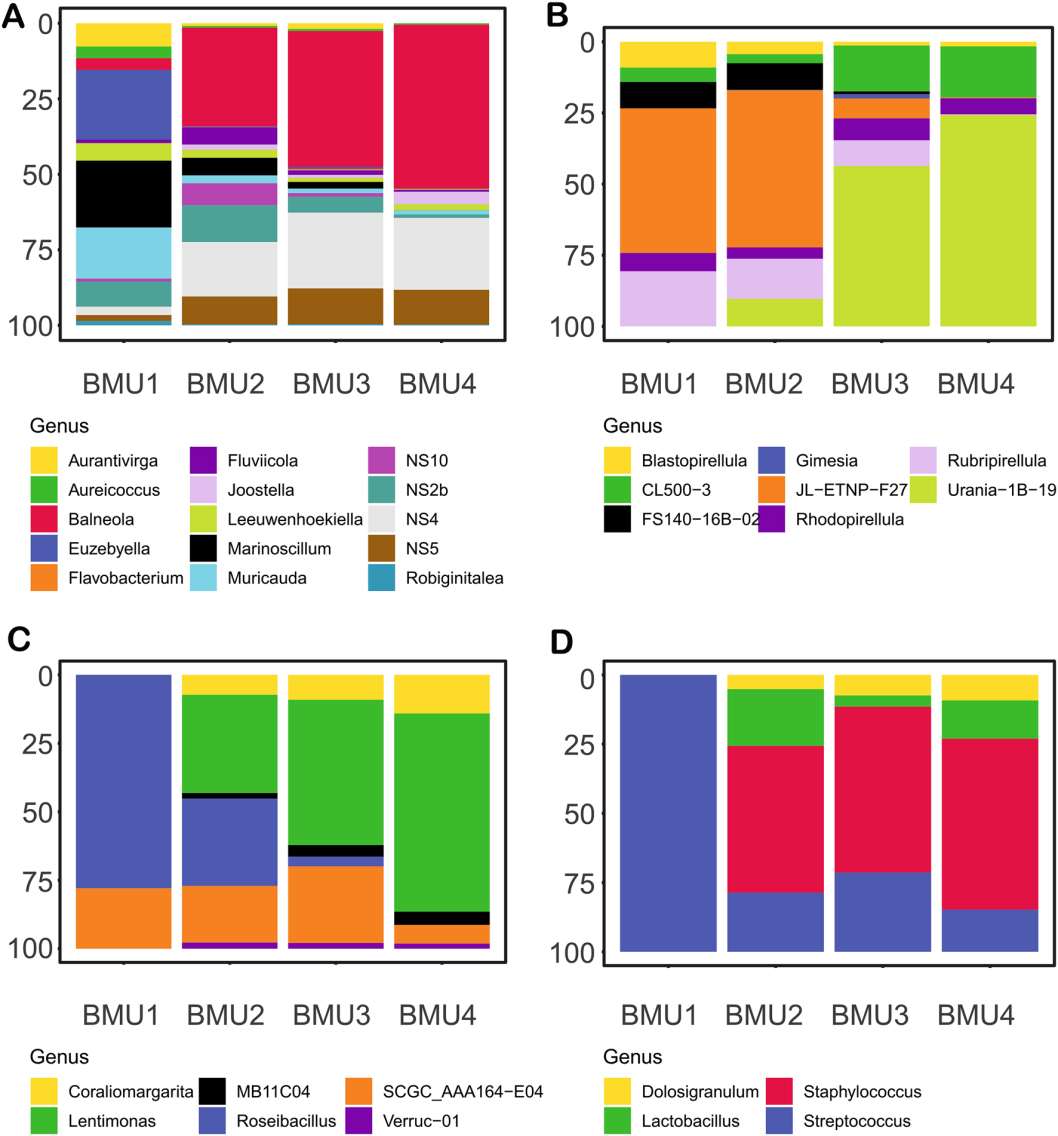
Characteristic genus contribution (**A**) Bacteroidota (**B**) Planctomycetota (**C**) Verrucomicrobia (**D**) Firmicutes. The figure was generated in R statistical software^35^ (https://cran.r-project.org/ , v. 3.6.3), using ggplot2 v. 3.3.3 package^36^, and they were assembled in Inkscape (https://inkscape.org/ , v. 0.92).

BCC-BMU1 included a single sample from station SAP3 from 100 m depth. It was unique because of the significant contribution of Nitrospinota (LS-NOB), the high contribution of Actinobacteriota, and the lower contribution of Cyanobacteria and Bacteroidota (Fig. 4). The differences were even more pronounced at genus level (Figs. 5, 6). Actinobacteriota were dominated by Curtobacterium and the uncultured Sva0996 lineage. An interesting pattern was observed for Bacteroidota that, despite the lower relative abundance, show higher diversity at the genus level, with a relatively equal contribution of all genera, e.g. *Mauricauda*, *Marinoscillum* or *Euzebyella*. Similarly, Alphaproteobacteria were very diverse in this BMU, with equal contributions of *Erythrobacter*, *Maricaulis*, *Martella*, uncultured OM75 clade, *Tistilia* and *Tistrella*. In contrast, Gammaproteobacteria were less diverse in this deep-water BMU than in the other units, with a higher contribution of uncultured UP05 lineage and genus *Woeseia* (Fig. 5). Planctomycetota were dominated by the uncultured JL-ENTP-F27 lineage, with a minor contribution of *Rubripirellula*, while Verrucomicrobiota was dominated by *Roseibacillus* with a minor contribution of the uncultured SCGC_AAA164-Eo4 lineage. *Prochlorococcus* was the dominant genus of Cyanobacteria.

BCC-BMU2 represents 23% of samples, mostly from the DCM layers (JP-66, JP-75, SAP3-78). This BMU appears transitional between the BCC-BMU1 and the other BCC-BMUs. This layer had the highest proportion of Cyanobacteria, represented in relatively equal proportions by *Synechococcus* and *Prochlorococcus* (Fig. 5). The contribution of Actinobacteriota was similar to that of the BCC-BMU1 (Fig. 5), but the relative abundance of *Curtobacterium* and the uncultured Sva0996 lineage decreased, and *Ca*. Actinomarina contributed almost 50% to this phylum. Bacteroidota were dominated by uncultured NS lineages and genus *Balneola* (Fig. 5). Within Alphaproteobacteria, there was a higher contribution of *Ascidiaceihabitans* and *Methylobacterium*, but no genus was dominant. A similar pattern was noticed within Gammaproteobacteria, which showed a relatively equal contribution of all the genera, e.g. *Ca*. Tenderia, *Dyella*, *Litoricola*, SUP05 lineage or *Woeseia*, and Verrucomicrobiota, where *Coraliomargarita* and *Lentimonas* co-dominated with *Roseibacillus* and the SCGC_AAA164-E04 lineage. In contrast, the composition of Planctomycetota was very similar to that of BCC-BMU1 (Fig. 6).

BCC-BMU3 describes the most frequent pattern, grouping the samples from the surface layer down to 65 m depth, regardless of the area. It is characterised by the dominance of Proteobacteria and Cyanobacteria followed by higher contributions of Bacteroidota and Actinobacteriota (Fig. 4). *Ca*. Actinomarina and *Curtobacterium* dominated Actinobacteriota, with a minor contribution of the uncultured Sva0996 lineage (Fig. 5). The proportion of genus *Balneola* and uncultured NS4 and NS5 lineages of Bacteroidota further increased in this BCC-BMU. *Ascidiaceihabitans* contributed most to Alphaproteobacteria, with higher proportions of *Ca*. Puniceispirillum, uncultured HIMB11 and OM75 clades, and *Paracoccus*. Within Gammaproteobacteria no genus dominated and the contribution of e.g. *Ca*. Tenderia, *Litoricola*, *Pseudoalteromonas*, and OM60/NOR5 lineage were similar (Fig. 5). The composition of Planctomycetota was very different from that of the BCC-BMUs described in the deeper samples, with a clear dominance of Urania-1B-19 and CL005 lineages. Marine *Synechococcus* was the dominant Cyanobacterium, followed by *Cyanobium* and *Prochlorococcus* (Fig. 5).

Finally, BCC-BMU4 describes 32% of the observed samples from surface layers down to 40 m. This pattern is similar to that of the BCC-BMU3, but with a lower contribution of Cyanobacteria and a higher contribution of Proteobacteria and Bacteroidota (Fig. 4). The composition of most phyla at a finer taxonomic level was very similar to that of BCC-BMU3, but with lower proportions of the genera that dominated in BCC-BMU1 and BCC-BMU2 (Figs. 5, 6). Actinobacteriota were dominated by *Ca*. Actinomarina and *Curtobacterium*, but the uncultured Sva0996 lineage was absent, while *Nocardioides* and *Rhodococcus* showed increased proportions. The composition of Bacteroidota was almost identical to that of BCC-BMU3, with a visibly lower proportion of NS2b lineage. Alphaproteobacteria showed higher proportions of *Aurantimonas*, *Croceicoccus*, *Erythrobacter* and *Marivivens*, with Gammaproteobacteria of *Alteromonas* and *Litoricola* displayed the same pattern (Figs. 5, 6). A further increase of Urania-1B-19 lineage (Planctomycetota), *Lentimonas*, *Coraliomargarita* (Verrucomicrobia) and *Synechococcus* (Cyanobacteria) was observed compared to BCC-BMU3 (Figs. 5, 6).

### Bacterial community diversity patterns

**Figure 7:**
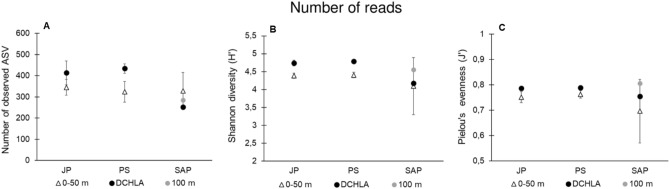
(**A**) Number of observed ASV. (**B**) Values of Shannon diversity index and (**C**) Values of Pielou’s evenness index at Jabuka Pit (JP), Palagruža Sill (PS) and South Adriatic Pit (SAP). Average values ± standard deviations (error bars) from layers are shown.

The rarefaction analysis indicated that this sequencing depth was sufficient to describe the diversity of bacteria in the investigated areas of the Adriatic Sea (Fig. S2). The average values of observed ASV numbers, Shannon diversity index (H′) and Pielou’s evenness (J′) showed the highest values at the DCM depth in the Jabuka Pit and Palagruža Sill, while at SAP the highest diversity and evenness was determined at 100 m depth and the highest number of ASVs was observed in the surface layer (0–50 m) (Fig. 7). All diversity indexes correlated with Chl *a* concentrations (approximated with fluorescence measurements): H′ (Spearman correlation: r = 0.45 n = 22, P < 0.05), J′ (r = 0.21, n = 22, p < 0.05) and number of ASVs (r = 0.56, n = 22, p < 0.05), indicating the relationship between bacterial diversity and phytoplankton biomass.

## Discussion

Environmental conditions affect both the rate of microbial processes and the composition of microbial communities in aquatic environments^47–49^. However, detailed knowledge about these relationships still lags behind data available for larger organisms, such as zooplankton or even microphytoplankton. One of the reasons is that the enormous bacterial diversity can be adequately described only with high-throughput sequencing methods that have been used in microbial ecology research for only about 15 years. Although huge progress has been made in describing microbial communities in the ocean^50^, bacterial diversity in many smaller basins is less known.

Here we present the first data of bacterial community composition in three open water basins of the oligotrophic Adriatic Sea, Jabuka Pit, Palagruža Sill and South Adriatic Pit. The data was obtained from a survey conducted in late summer 2016, and is associated with environmental parameters. During this survey the average values of SRP were < 0.1 µmol L^−1^ (N/P and Si/P were > 22), while total inorganic nitrate was < 1 µmol L^−1^ (N/P was < 10), suggesting that this area was generally phosphorus and nitrogen-limited. These values are typical of this region^17,29^, indicating that our results are applicable to wider contexts than this snap-shot study. Moreover, it is well-known that under such nutrient-poor conditions in marine ecosystems the microbial food web becomes the dominant pathway for carbon flux^51^. An important novelty of our study is that we used the Neural gas method to group samples with similar picoplankton characteristics, which subsequently allowed us to identify the most important environmental factors affecting their distribution. The algorithm is suitable for detecting anomalies and outlier data; in this survey, we had an unequal number of samples from the different basins. Thus, by using Neural gas method, we overcame the limitations of our research.

During thermal stratification and oligotrophic conditions, values of bacterial abundances and production were typical of the open Adriatic^11,12,14,15^. LNA bacteria were predominant in the P-limited epipelagic environment, which reflects their adaptation to nutrient-poor conditions^52^. Similarly, a general prevalence of *Synechococcus* cells within the picoautotrophic community, especially in the shallow epipelagic layer, was noted for P-depleted environments^11,13,14,53,54^. In contrast, HNA bacteria dominated in the upper N-limited layer and also in the deeper waters enriched with nutrients, which agrees with previous observations from the oligotrophic open sea station during the summer^55^ and from the open Adriatic Sea^11,14,15^. Moreover, our results suggest that temperature increases have a positive impact on the picoplankton community in this P and N limited open sea area. On a seasonal scale, it was determined that the bacterial community was under a positive influence of temperature^10^. We also observed a negative relationship between picoplankton and salinity in at least 81% of the dataset, as discovered recently in the central Adriatic Sea^21^. Furthermore, our analysis of relationships between picoplankton and environmental characteristics pointed out that all observed parameters were more differentiated by depth than by area (Fig. 2B). The highest values of bacterial abundance and production were distributed in the shallow epipelagic layer, while the highest diversity was found in the deeper layer (Fig. 7), thus confirming the findings of previous research^1,56^. Korlević et al.^19^ have linked the increase in diversity with deep convection mixing of the seawater in the Adriatic Sea, which transports nutrients to the euphotic layer and triggers phytoplankton blooms. On the other hand, higher bacterial diversity was also shown to correlate with lower bacterial abundance and production^19,57^, as observed here (Fig. 6).

According to sequence data, *Prochlorococcus* was present in high proportions below 50 m depth (Fig. 3F), which may be attributed to the presence of a low light-adapted ecotype^58^. However, flow cytometry data confirms its prevalence only in sample SAP3 at 100 m, but absolute abundances of *Synechoccocus* exceeded that of *Prochlorococcus* in most samples (Table S2). An interesting observation is the presence of *Cyanobium*-related sequences. *Cyanobium* was originally established as an exclusively freshwater genus (based on the reference strain *C. gracile* PCC 6307 and its relatives), but recently several marine representatives of the genus were also isolated. It is also important to note that the primers used in this study poorly cover the SAR11 clade^30^, which dominates bacterial communities in the seas and oceans^59^, contributing up to 50% in most oligotrophic areas^60^. This clade is also dominant in the Mediterranean Sea^61^ and the southern Adriatic sub-basin^62^. Therefore, it is highly likely that the proportion of SAR11 clade is underestimated in our study.

To link the changes in the composition of the bacterial community and environmental features we combined the results of 16S rDNA amplicon sequencing with Neural gas analysis. Recently, it has been shown that although amplicon sequencing may not accurately reflect the relative abundance of specific phylotypes, it allows for reliable ecological interpretations at the community level^63^. Our results clearly showed that depth was the key factor differentiating bacterial community composition in all investigated areas: Jabuka Pit, Palagruža Sill and South Adriatic Pit. Numerous studies have shown that bacterial community structure changes with depth in different oligotrophic systems, such as the Pacific and Atlantic^64^, as well as the Mediterranean Sea^65,66^ suggesting that vertical stratification is a very important factor^65,67^.

Neural gas analysis revealed four diversity patterns of bacterial community composition (BCC-BMUs) during the summer stratification.

The first pattern (BCC-BMU1) was found at 100 m depth, the coldest investigated layer, not limited by N or P (PIC-BMU2). Although it included only a single sequenced sample, it is possible that similar bacterial communities would have been found in other deep-water (> 100 m) samples of the PCI-BMU2. The majority of reads were attributed to the uncultured SUP05 lineage of Gammaproteobacteria (Fig. 5). These chemoautotrophic sulfur-oxidizing microbes are ubiquitous in diverse marine minimum oxygen zones^68,69^. Moreover, what makes this pattern special with regards to the shallower layers is the higher contribution of phylum Nitrospinota, with the majority of reads attributed to the LS-NOB lineage. Nitrospinae uses the energy provided by nitrite oxidation to assimilate bicarbonate, which is a vital function for dark-ocean carbon fixation^70^. This pattern also includes increased relative abundances of Chloroflexota (Fig. 3A), represented by the uncultured SAR202 lineage. These heterotrophic bacteria are an important component of bathypelagic bacterial communities, and as potential sulfite-oxidizers may play a specific role in the sulfur cycle^71^. Another important uncultured heterotroph in this BCC-BMU belonged to the marine group Sva0996 of Actinobacteriota (Fig. 5). They display a close relationship with organic matter^72^ and have an important role in dissolved organic nitrogen cycling in the ocean^73^. Based on the total prokaryote numbers in PIC-BMU1, these groups did not make up abundant populations, despite nutrient concentrations, which could be due to low temperature (Table 1).

The second pattern of bacterial community composition (BCC-BMU2) was found in the DCM layer. This layer was characterised by an increased abundance of cyanobacteria (Fig. 2, Table S2). In terms of bacterial community composition, a higher proportion of lineages that probably participated in the decomposition of organic matter secreted at these depths by cyanobacteria were observed, e.g. *Ca.* Actinomarina and Sva0996 of Actinobacteriota, *Baleneola* and uncultured lineages NS2b, NS4 and NS5 of Bacteroidota, and uncultured Planctomycetota from the JL–ETNP–F27 lineage (Fig. 6). In general, this BCC-BMU was intermediate between the BCC-BMU1 associated with cold, nutrient-rich waters (PIC-BMU2) and the BCC-BMU3 that is characteristic of P and N limited waters around the thermocline (PIC-BMU4 and 5).

Two patterns, BCC-BMU3 and BCC-BMU4, were associated mainly with shallow epipelagic layers (PCI-BMU 4 and 5). BCC-BMU3 was intermediate between BCC-BMU2 and BCC-BMU4, which may result from the fact that BCC-BMU3 seems to be related with the thermocline, as seawater temperatures > 15.6 °C were recorded, while BCC-BMU4 includes mainly samples above the thermocline with seawater temperatures > 18 °C (Table S1). In general, the dominant bacteria in these BMUs are known to be associated with summer phytoplankton blooms or to degrade labile organic matter, e.g. alphaproteobacterium *Ascidiaceihabitans*^74^ and Gammaproteobacterial *Pseudoalteromonas* and *Pseudomonas* (Fig. 5). However, the genus *Alteromonas* (Gammaproteobacteria), also an important bacterium for the degradation of organic matter, was important only in BCC-BMU4.

Other phyla that substantially contributed to relative abundances in these BMUs were Bacteroidota and Actinobacteriota (Fig. 4). Phylum Bacteroidota is known to represent one of the most abundant bacterial groups in coastal environments, exhibiting a free-living or particle-attached lifestyle^61,75,76^. They are believed to play a major role in the degradation of organic matter in the investigated area. Members of this phylum were also abundant in the southern Adriatic sub-basin, with more operational taxonomic units at the station with the highest content of total suspended matter and particulate organic carbon^62^. Furthermore, this phylum showed the highest contribution in the shallow epipelagic layer, which agrees with^77^, who showed that relative abundances of Bacteroidota decrease with depth. We showed that this overall decrease was connected to change in their composition at finer taxonomic level, from the dominance of uncultured marine NS clusters to an increase of cultivated genera in the deepest layers, as already evidenced in the Mediterranean Sea^78^.

Noteworthy, the genera that are important for delineating different BCC-BMUs were not necessarily those with the highest relative abundance in the samples, but rather those whose contribution to bacterial communities differed most. The most conspicuous example is the alphaproteobacterial AEGEAN-169 lineage and gammaproteobacteria SAR86 clade, which contributed to all the samples (Fig. 3), but were not identified as important in any of the BCC-BMUs (Fig. 5). This may be due either to the high plasticity of such lineages, or to their functional diversity that cannot be recognized at the level of 16S rRNA genes.

In general, our results indicate relationships between environmental factors, picoplankton abundance, production and bacterial community composition, pointing to the importance of the species sorting processes in the open Adriatic Sea, as commonly observed in aquatic environments^79^. However, some exceptions were observed. For instance, environmental conditions were rather unique at 10 m depth in Jabuka Pit, where N-limited conditions could result in increased abundances of prokaryotes, HNA and AAP bacteria (Fig. 2, Table 1). Nevertheless, BCC composition in this sample did not differ substantially from the other samples in Jabuka Pit or BCC-BMU3 (Fig. 3), thus indicating the importance of other ecological variables in shaping bacterial communities in the area.

## Conclusions

The picoplankton community displayed heterogenic response to different environmental factors in the open sea areas of the central and southern Adriatic Sea, throughout the stratified water column.

The use of Neural gas analysis allowed us to determine associations between selected environmental parameters and observed picoplankton variables, bacterial community structure and profiles.

Our results provide a novel insight into factors affecting picoplankton in this area. They revealed that picoplankton and bacterial community composition were more associated with the depth rather than investigated area. Furthermore, an increase in temperature correlated positively with the picoplankton community (heterotrophic bacteria, HNA%, LNA%, *Synechococcus*, *Prochlorococcus*, picoeukaryotes, AAP, bacterial production) in this N and P limited environment. BCC showed the dominance of Proteobacteria and Cyanobacteria, followed by Bacteroideota and Actinobacteriota phyla, whereas changes at finer taxonomic levels were related to distinct environmental conditions at different depths. Future research should focus on high-frequency sampling of bacterial community composition, together with environmental parameters, to obtain a clearer representation of ecological patterns.

## Supplementary Information

Supplementary Information.

## Data Availability

The data supporting the findings of this study are available on request from the corresponding author Danijela Šantić, segvic@izor.hr, Institute of Oceanography and Fisheries.
