# Supplementary material for: Artificial neural network analysis of microbial diversity in the central and southern Adriatic Sea
Source: Sci Rep. 2021 May 27;11:11186. doi: 10.1038/s41598-021-90863-7 (PMC8159981; doi:10.1038/s41598-021-90863-7)
Supplement: Supplementary file 1 — Supplementary Information. [file 41598_2021_90863_MOESM1_ESM.pdf]

Supplementary Information

Artificial neural network analysis of microbial diversity in the Central and Southern Adriatic Sea

Danijela Šantić*[1], Kasia Piwosz[2], Frano Matić[1], Ana Vrdoljak Tomaš[1], Jasna Arapov[1], Jason Lawrence Dean[3], Mladen Šolić[1], Michal Koblížek[3,4], Grozdan Kušpilić[1], Stefanija Šestanović[1]

1) Institute of Oceanography and Fisheries, Šetalište Ivana Meštrovića 63, 21000 Split, Croatia

2) National Marine Fisheries Research Institute, Kołłątaja 1, 81-332 Gdynia, Poland

3) Centre Algatech, Institute of Microbiology of the Czech Acad. Sci., 379 81 Třeboň, Czech Republic

4) University of South Bohemia, Faculty of Science, Branišovská 1760, České Budějovice, Czech Republic

*Corresponding author: Danijela Šantić, Institute of Oceanography and Fisheries, POB 500, 21000 Split, Croatia; Phone: 385-21-408006, Fax: 385-21-358650, e-mail: segvic@izor.hr

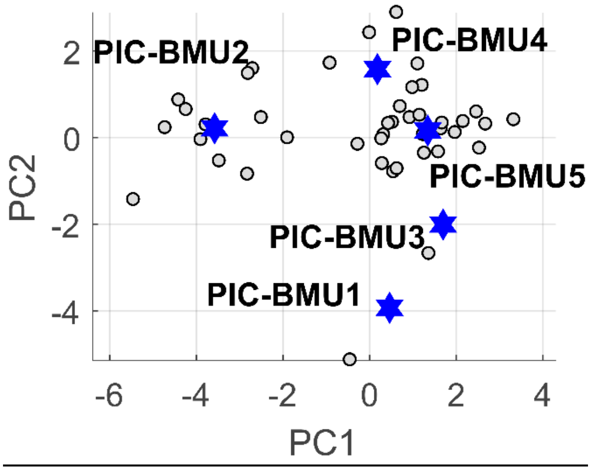

Figure S1. Projection of input data (grey dots) and best-matching units from neural gas (blue stars) on main input data principal components. The software MATLAB. version 7.10.0 (R2018). Natick, Massachusetts: The MathWorks Inc. (2018) was used to generate the figure.

Table S1. Environmental parameters values

Area	Station and depth	Tempe-rature (ºC)	Salinity	NO_3^- (μM)	NO_2^- (μM)	NH_4^+ (μM)	N-organic (μM)	PO_4^{3-} (μM)	P-organic (μM)	SiO_4^{2-} (μM)	O_2 (mLL^{-1})
South Adriatic Pit	SAP1-0m	22.99	38.60	0.80	0.14	0.49	4.79	0.05	0.28	2.46	5.36
	SAP1-5m	23.29	38.70	0.21	0.07	0.41	8.25	0.05	0.13	1.17	5.33
	SAP1-10m	23.46	38.74	0.21	0.06	0.44	6.01	0.03	0.13	1.09	5.25
	SAP1-20m	23.36	38.76	0.21	0.05	0.59	6.28	0.05	0.16	0.95	5.19
	SAP1-50m	18.32	38.82	0.21	0.05	0.49	5.47	0.02	0.14	0.74	6.20
	SAP1-105m	16.15	38.96	0.66	0.35	0.51	4.55	0.12	0.26	1.41	5.39
	SAP2-0m	23.20	38.77	0.21	0.02	0.35	4.23	0.03	0.12	0.94	5.26
	SAP2-5m	23.07	38.76	0.21	0.02	0.39	4.73	0.04	0.22	0.96	5.20
	SAP2-10m	23.06	38.77	0.21	0.05	0.43	5.13	0.03	0.08	0.94	5.27
	SAP2-20m	23.06	38.78	0.21	0.02	0.45	4.56	0.03	0.10	1.00	5.27
	SAP2-50m	16.28	38.85	0.21	0.09	0.57	4.95	0.03	0.11	1.13	6.08
	SAP2-100m	15.27	38.93	1.56	0.06	0.41	4.41	0.06	0.08	1.28	5.38
	SAP2-200m	14.34	38.79	2.04	0.07	0.51	3.85	0.09	0.08	1.39	5.55
	SAP2-300m	14.23	38.78	2.44	0.12	0.47	5.74	0.13	0.20	1.59	5.43
	SAP3-0m	22.32	38.78	0.21	0.05	0.46	5.00	0.02	0.14	1.17	5.31
	SAP3-5m	22.33	38.85	0.21	0.02	0.42	4.59	0.06	0.04	1.06	5.39
	SAP3-10m	22.33	38.85	0.21	0.02	0.62	4.46	0.02	0.12	1.14	5.30
	SAP3-20m	18.81	38.81	0.21	0.02	0.56	5.59	0.05	0.09	1.13	6.50
	SAP3-50m	15.82	38.93	0.21	0.05	0.59	4.37	0.04	0.06	1.06	5.97
	SAP3-100m	15.20	38.93	1.75	0.20	0.59	4.40	0.11	0.11	1.39	5.35
	SAP3-200m	14.35	38.80	2.43	0.05	0.41	4.22	0.07	0.15	1.62	5.50
	SAP3-1190m	13.30	38.72	3.78	0.05	0.44	4.39	0.27	0.03	5.72	5.18
Palagruža Sill	PS1-0m	22.62	38.75	0.86	0.16	0.19	10.38	0.05	0.17	1.27	5.55
	PS1-5m	22.49	38.91	0.21	0.02	0.10	4.39	0.09	0.03	0.97	5.45
	PS1-10m	22.47	38.91	0.21	0.05	0.19	5.75	0.08	0.03	0.96	5.66
	PS1-20m	22.21	38.88	0.21	0.06	0.13	0.94	0.10	0.01	1.02	5.81
	PS1-50m	16.17	38.89	0.21	0.06	0.11	8.93	0.03	0.12	0.82	6.00
	PS1-100m	15.48	38.93	0.82	0.12	0.09	7.98	0.04	0.08	1.16	5.45
	PS1-175m	13.62	38.72	2.52	0.08	0.08	6.90	0.10	0.11	2.79	5.32
	PS2-0m	22.20	38.71	0.21	0.07	0.13	8.62	0.02	0.10	1.06	5.52
	PS2-5m	21.83	38.85	0.21	0.05	0.09	9.71	0.06	0.09	0.99	5.47
	PS2-10m	21.79	38.84	0.21	0.02	0.07	9.15	0.01	0.11	1.00	5.54
	PS2-20m	18.15	38.83	0.21	0.06	0.12	12.74	0.01	0.15	1.03	6.87
	PS2-50m	15.60	38.90	0.21	0.02	0.05	10.99	0.03	0.08	1.09	6.12

	PS2-105m	14.89	38.87	1.36	0.08	0.14	9.26	0.07	0.06	1.16	5.38
Jabuka Pit	JP-0m	21.76	38.70	0.95	0.07	0.50	2.97	0.01	0.19	0.95	5.40
	JP-5m	21.61	38.69	0.21	0.02	0.67	9.57	0.03	0.15	0.87	5.42
	JP-10m	21.58	38.70	0.21	0.07	0.04	10.43	0.02	0.13	0.87	5.38
	JP-20m	20.52	38.73	0.21	0.06	1.09	10.23	0.01	0.17	1.05	5.92
	JP-50m	15.99	38.92	0.21	0.04	0.70	7.40	0.08	0.05	0.64	5.98
	JP-100m	15.04	38.87	0.79	0.13	0.83	7.34	0.10	0.08	1.24	5.43
	JP-200m	13.21	38.69	0.91	0.16	0.73	7.89	0.06	0.14	1.17	5.46
	JP-260m	12.94	38.68	1.48	0.07	0.75	7.39	0.13	0.02	1.47	5.42

Table S2. Abundances of aerobic anoxygenic phototrophs-AAP, AAP%, total prokaryotes-TP, High nucleic acid content bacteria-HNA%, Low nucleic acid content bacteria-LNA%, *Synechococcus*-SYN, *Prochlorococcus*-PROCHL, picoeukaryotes-PE, heterotrophic nanoflagellates-HNF, bacterial production-BP

Area	Station - depth	AAP x 10^{4} (cell mL^{-1})	AAP (%)	TP x 10^{6} (cell mL^{1})	HNA (%)	LNA (%)	SYN x 10^{3} (cell mL^{-1})	PROCHL x 10^{3} (cell mL^{1})	PE x 10^{3} (cell mL^{-1})	HNF x 10^{3} (cell mL^{-1})	BP x 10^{4} (cell h^{-1} mL^{-1})
South Adriatic Pit	SAP1-0m	2.11	5.85	0.29	44.20	55.80	19.99	1.20	0.99	1.17	0.06
	SAP1-5m	2.21	5.89	0.30	43.10	56.90	23.86	1.65	1.09	1.19	0.07
	SAP1-10m	1.55	4.48	0.27	46.80	53.20	14.48	3.94	1.09	1.17	0.09
	SAP1-20m	1.27	4.68	0.24	45.40	54.60	11.80	3.00	0.92	1.00	0.08
	SAP1-50m	1.23	3.39	0.25	45.90	54.10	12.04	0.37	1.06	1.06	0.08
	SAP1-105m	0.46	2.30	0.19	49.50	50.50	1.15	0.49	0.20	0.40	0.05
	SAP2-0m	1.27	4.80	0.23	50.50	49.50	5.21	2.02	0.70	0.86	0.07
	SAP2-5m	1.49	5.56	0.24	50.60	49.40	5.09	1.92	0.61	0.95	0.06
	SAP2-10m	1.52	5.63	0.20	43.80	56.20	4.93	1.92	0.79	0.79	0.05
	SAP2-20m	1.28	4.43	0.21	47.60	52.40	5.13	2.08	0.71	0.87	0.05
	SAP2-50m	0.36	1.61	0.26	38.50	61.50	7.66	0.64	1.01	1.00	0.08
	SAP2-100m	0.21	1.03	0.13	46.00	54.00	2.03	0.34	0.49	0.33	0.04
	SAP2-200m	/	/	0.08	53.10	46.90	0.32	0.25	0.07	0.30	0.06
	SAP2-300m	/	/	0.07	56.50	43.50	0.28	0.21	0.04	0.22	0.02
	SAP3-0m	1.08	6.22	0.18	45.40	54.60	2.51	1.81	0.81	0.93	0.04
	SAP3-5m	0.93	5.05	0.18	46.40	53.60	2.39	1.77	0.79	1.24	0.07
	SAP3-10m	1.23	6.83	0.18	43.70	56.30	3.01	1.80	0.88	1.13	0.05
	SAP3-20m	1.16	4.15	0.24	40.00	60.00	1.03	1.55	0.83	1.44	0.12
	SAP3-50m	0.36	1.52	0.28	54.30	45.70	9.72	0.34	1.33	1.48	0.09
	SAP3-100m	0.09	0.60	0.09	57.10	42.90	0.12	0.24	0.46	0.40	0.03
	SAP3-200m	/	/	0.08	52.20	47.80	0.08	0.16	0.07	0.14	0.03
	SAP3-1190m	/	/	0.05	65.60	34.40	0.12	0.26	0.01	0.05	0.05
Palagruža Sill	PS1-0m	1.38	5.34	0.28	51.00	49.00	5.50	1.59	0.87	0.97	0.36
	PS1-5m	1.40	6.67	0.25	47.20	52.80	5.46	2.12	0.92	1.21	0.06
	PS1-10m	1.32	5.74	0.46	73.20	26.80	5.37	1.45	0.90	0.62	0.06
	PS1-20m	1.46	4.96	0.26	46.40	53.60	6.15	1.63	0.93	1.18	0.07
	PS1-50m	1.44	4.94	0.24	48.10	51.90	9.78	0.39	0.76	0.70	0.07
	PS1-100m	0.28	1.24	0.13	46.20	53.80	1.56	0.50	0.42	0.20	0.03
	PS1-175m	0.09	0.42	0.17	53.00	47.00	0.62	0.30	0.08	0.23	0.06
	PS2-0m	1.82	5.07	0.28	44.20	55.80	9.00	1.57	0.99	0.97	0.05

	PS2-5m	1.59	4.56	0.32	43.60	56.40	8.62	1.99	0.97	1.45	0.09
	PS2-10m	1.73	4.87	0.30	45.10	54.90	6.36	1.90	1.15	1.25	0.07
	PS2-20m	/	/	0.33	37.80	62.20	11.09	0.32	1.31	1.65	0.09
	PS2-50m	2.23	4.44	0.32	37.80	62.20	0.31	0.29	0.22	1.11	0.08
	PS2-105m	0.36	1.79	0.10	45.60	54.40	19.29	2.00	0.88	0.23	0.02
Jabuka Pit	JP-0m	1.23	5.16	0.36	49.10	50.90	9.29	1.06	0.69	1.60	0.04
	JP-5m	1.19	4.84	0.31	51.80	48.20	10.19	0.63	0.75	1.28	0.08
	JP-10m	1.76	5.85	0.44	57.40	42.60	8.20	1.02	0.79	1.10	0.13
	JP-20m	1.82	4.92	0.37	42.20	57.80	10.09	0.72	1.01	1.59	0.07
	JP-50m	1.00	3.16	0.34	40.30	59.70	14.37	0.70	0.88	1.14	0.03
	JP-100m	0.47	1.78	0.24	52.60	47.40	1.03	0.92	0.74	0.60	0.03
	JP-200m	0.49	1.93	0.20	58.60	41.40	0.45	0.21	0.66	0.51	0.02
	JP-260m	0.19	0.94	0.21	57.70	42.30	0.64	0.32	0.21	0.57	0.02

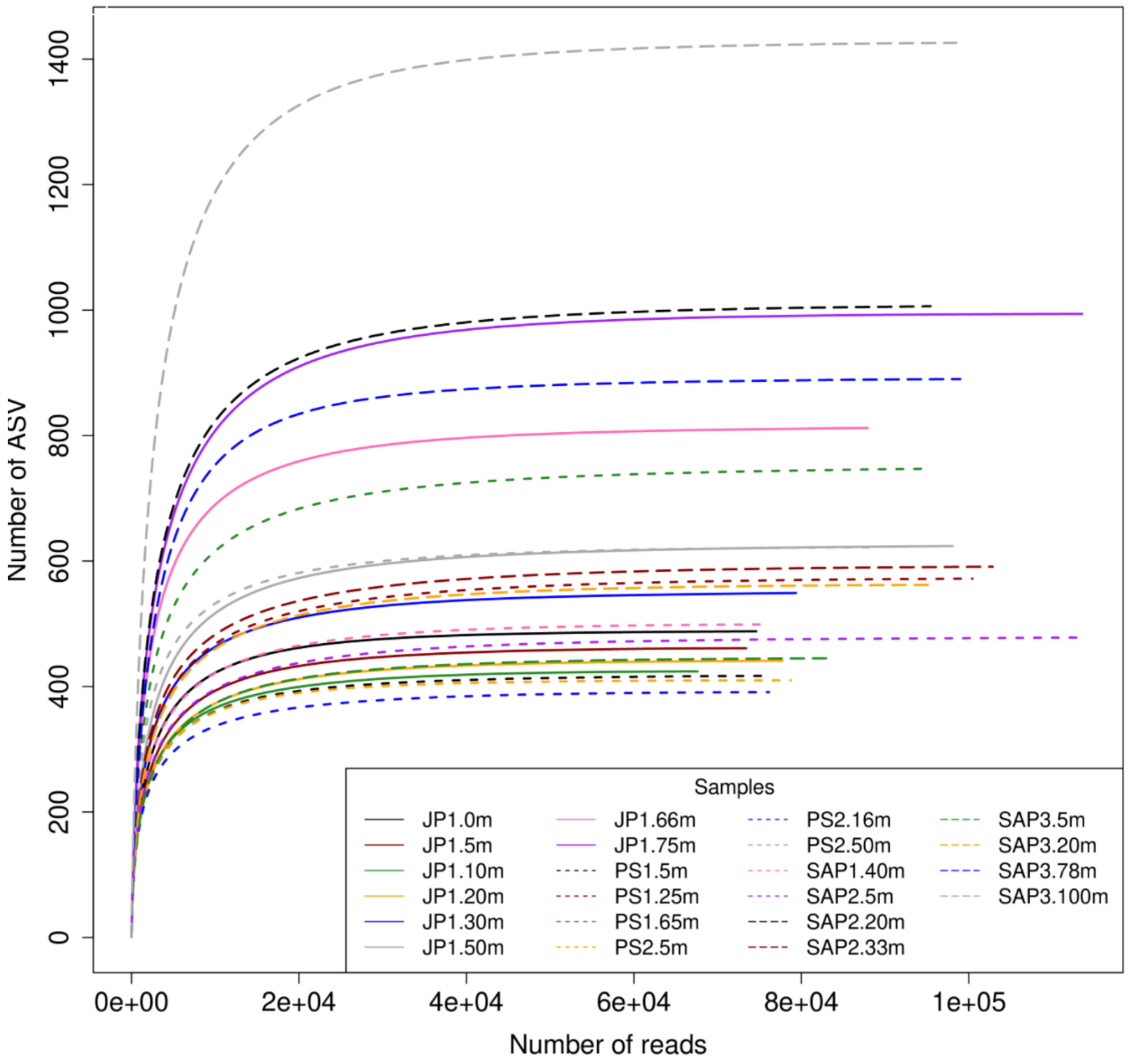

Figure S2. Species accumulation curve in the samples (lower X-axis), and rarefaction curve for all samples combined (upper X-axis)
